# Evaluation of Anxiety Levels in Patients Undergoing Intravitreal Injections and Associated Risk Factors Related to the Disease

**DOI:** 10.1155/2020/4375390

**Published:** 2020-10-19

**Authors:** Juan-Carlos Herranz-Heras, Almudena de-Pablo-Cabrera, Beatriz Alonso-Martín, Beatriz de-Lucas-Viejo, Marta de-Castro-Liébana, Enrique Mencía-Gutiérrez, Manuel-Jesús Ferro-Osuna, Carmen Romero, Javier Sambricio

**Affiliations:** ^1^Ophthalmology Department, 12 de Octubre Hospital, Complutense University, Madrid 28041, Spain; ^2^Biostatistical Research Institute (i + 12), 12 de Octubre Hospital, Complutense University, Madrid 28041, Spain

## Abstract

**Purpose:**

To analyze patients' anxiety levels using the Visual Analog Scale for Anxiety (VASA), in regard to intravitreal injection treatment and to determine possible associated risk factors related to the disease and treatment characteristics.

**Methods:**

Cross-sectional observational study with consecutive sampling of patients who were going to receive an intravitreal injection. Subjects completed the VASA prior to the procedure, and afterwards, their data were collected from the electronic medical history. Analysis was performed through a linear regression model.

**Results:**

Fifty-five men and forty-seven women were enrolled. The mean age was 73.9 ± 12.4 years (mean ± standard deviation (SD)), and the mean ± SD of previous injections was 12.8 ± 12. The most frequent pathologies found were age-related macular degeneration with 46.1% and diabetic macular edema with 36.3%. The median of anxiety levels measured in millimeters (mm) was 16 (interquartile range: 0–48). In univariate models, women presented a mean of 10.8 mm of anxiety more than men (*p*=0.03). The adjusted multivariate analysis demonstrated that younger patients declared higher anxiety levels (*p*=0.036). No significant association was found between the best corrected visual acuity (BCVA) on the day of the injection, the change in BCVA since the beginning of the treatment or the number of injections received, and the registered anxiety levels.

**Conclusions:**

Sex and age may have an influence on anxiety levels. BCVA and the number ofinjections received did not seem to have an influence on our patients anxiety levels.

## 1. Introduction

Age-related macular degeneration (AMD) and diabetic macular edema (DME) are the main causes of irreversible blindness worldwide. It is estimated that the prevalence of DME in diabetic patients is around 7%, and the prevalence of AMD is approximately 30% of the population over 75 years of age [1, 2]. Both pathologies have a great impact on the quality of life of patients, they entail a heavy burden for both healthcare systems and patients, and they have a high healthcare cost [3, 4].

It is well known that vascular endothelial growth factor (VEGF) plays an important role in the pathophysiology of these two diseases, as well as in others that cause central vision loss [5]. The inhibition of VEGF is the main treatment for these pathologies, and all VEGF inhibitors are administered in the form of intravitreal injections [6, 7]. Another therapeutic strategy for DME or macular edema secondary to other diseases, such as retinal vein occlusion (RVO), is the intravitreal injection of dexamethasone implants [8]. All of these drugs have been proven to be safe and effective for the treatment of these pathologies; however, they do require a heavy burden of periodic intravitreal injections, often long term. There are different therapeutic strategies, mainly in the treatment for AMD. There is a monthly schedule, a pro re nata (PRN) strategy and a treat-and-extend (T&E) strategy. In the PRN and T&E strategies, patients receive a series of monthly loading injections. After these loading injections, in the PRN approach, patients have regular visits and receive an injection when the activity of the disease is noticed, and in the T&E schedule, when criteria indicating no disease activity are met, patients receive an anti-VEGF injection, and the treatment interval is extended, usually by two weeks [6–8].

Patient anxiety regarding a medical procedure, especially a surgical one, is a problem that has been well-known for decades. Patients often feel anxious due to fear of the procedure itself or the pain related to it, among other aspects [9]. Some studies claim that between 20% and 30% of the general population experience high levels of anxiety regarding their health, and these levels can rise significantly when patients are facing a medical procedure [10]. Moreover, it has been demonstrated that elevated levels of anxiety can have a negative impact on the results of the procedure, as well as on the patient's feelings of satisfaction [11]. The objective of this study is to evaluate the levels of anxiety produced by intravitreal injections and to determine possible associated risk factors.

In our study, we intended to correlate anxiety levels with factors related to the disease. Our hypothesis was that factors related to the disease patients' anxiety was not only related to the intravitreal injection itself, but also suffering from factors related to the disease might have an impact on their anxiety levels. Therefore, patients who had lower initial BCVA, whose BCVA deteriorated since diagnosis, those who had received a great number of prior injections, or those who needed changes in treatment due to inefficacy of previous treatments might have higher levels of anxiety. To our knowledge, no previous studies have studied these factors.

## 2. Materials and Methods

This study adhered to the principles of the Declaration of Helsinki and was approved by the Ethics Committee of 12 de Octubre Hospital, Madrid, Spain.

### 2.1. Study Design

A cross-sectional, consecutive, observational, and noninterventional study was conducted including patients who were treated in 12 de Octubre Hospital, Madrid, Spain, between February and March 2018. All the patients included were diagnosed with AMD, DME, or macular edema secondary to RVO or other macular pathologies and were going to receive an intravitreal injection of bevacizumab, ranibizumab, or aflibercept.

The inclusion criteria were patients over the age of 18 years who had been diagnosed with one of the previously mentioned pathologies requiring treatment with intravitreal injections. The exclusion criteria established were the presence of cognitive impairment and current regular treatment with anxiolytic or antidepressant medication.

Upon arrival to the injection room, the aims and methods of the study were explained to patients who met the inclusion criteria and none of the exclusion criteria, and written informed consent was obtained from those who wished to participate. Next, a self-administered survey was given for them to complete, in the presence of the interviewer, before they received the intravitreal injection.

### 2.2. Procedure

The survey included two questions regarding their treatment and the Visual Analog Scale for Anxiety (VASA). The questions were (1) how many injections have you previously received? and (2) do you know the possible risks of the injection? The VASA consisted of a straight horizontal line 100 millimeters (mm) long, with perpendicular crossing small vertical lines every 10 mm. On the far left end, scored as 0, was “not anxious at all,” and on the far right end, scored as 100, was “extremely anxious.” The patients marked their subjective appreciation of the level of anxiety they were experiencing at that moment in relation to the procedure with a vertical line on the scale from 0 to 100. The patients were asked to state their answer out loud when completing the survey so that the interviewer could make sure it was being well interpreted. The placement of the mark made by the patient was then measured in mm from the left end for its subsequent analysis. To set a better clinical understanding of the results of the VASA, we categorized the results into four quartiles defining high-anxious patients those who were included in the third quartile and very-high-anxious patients those who were included in the fourth quartile. Moreover, we established a difference of 10 mm or more between groups as a difference clinically relevant.

After the survey was completed, all patients underwent a best-corrected visual acuity (BCVA) examination in both monocular and binocular vision and measured in a decimal scale. When the BCVA was lower than 0.05, it was given the value 0 for statistical analysis. The data for improvement of visual acuity since the beginning of treatment were obtained by comparing this measurement of BCVA with the one registered at the moment of diagnosis of the disease.

No longer than 15 minutes after the completion of the survey and examination for visual acuity, the patients received the intravitreal injection as programmed. The procedure was conducted in a clean room following the usual antiseptic and anesthetic conditions, including topical anesthesia and disinfection with 5% ophthalmic povidone-iodine. After the injection is performed using the straight injection technique with a 30-gauge needle, the absence of adverse events is verified and symptoms of alarm are explained to the patient before their departure.

In our center, after the diagnosis of macular pathology that requires intravitreal injection of medication, the injection protocol is initiated the same day, and afterwards, the remaining required injections are programmed according to the patient's necessities. Often, the patient comes to the clinic only to receive treatment, without having a medical visit with their treating ophthalmologist. Intravitreal injections are performed by 9 different ophthalmologists in random order, depending on the service's organizational needs. The patient does not know beforehand who will be the ophthalmologist performing the intravitreal injection. In our sample, we included patients who received a programmed intravitreal injection as well as those receiving treatment for the first time.

Afterwards, the following data were obtained from electronic medical history: age, sex, diagnosed pathology in treatment, type of anti-VEGF administered and previous anti-VEGF if applicable, number of eyes treated, number of injections received to date, improvement of BCVA in the treated eye, and improvement of binocular BCVA.

### 2.3. Sample Size

Power calculations indicated that a sample size of 100 participants was sufficient to detect a small effect size (*f*^2^ = 0.15) with a statistical power of 0.80. A total of 102 patients were included. These calculations were accomplished with the software program G^*∗*^Power 3.1 [12].

### 2.4. Statistical Analysis

Descriptive statistics were used with continuous variables (mean, standard deviation (SD), interquartile ranges (IR), etc.), and for categorical variables, absolute and relative frequencies were used.

The analysis of the factors that influence the level of anxiety, measured in mm, was performed employing a univariate linear regression model. The variables analyzed were age (decades), sex, initial binocular BCVA, improvement of binocular BCVA, treatment naïve vs. treatment experienced, real number of injections received, subjective number of injections received (as declared by the patient in the survey), changes in treatment since the beginning of the illness, number of eyes treated, anti-VEGF, and treated disease. Univariate linear regression models were performed analyzing anxiety as a dependent variable and the different factors mentioned above as independent variables. Afterwards, a multivariate regression model was performed in which all the factors with a *p* value <0.15 in the univariate analysis were included. This initial multivariate model was adjusted using the “backward” method until reaching the final fitted multivariate model. For all analyses, a *p* value <0.05 was considered as the limit to establish statistically significant differences.

All data were analyzed using SAS system version 9.4® (SAS Institute Inc., Cary, NC, USA).

## 3. Results

In this study, 102 patients were enrolled, 55 males (53.9%) and 47 females (46.1%). The mean age was 73.9 ± 12.4 years (mean ± SD). The pathologies for which the patients were receiving treatment were in decreasing order of frequency: AMD (46.1%), DME (36.3%), RVO (11.7%), and other pathologies such as macular telangiectasia type 1 or chronic serous chorioretinopathy (5.9%).

Over half of the patients enrolled (58.8%) were receiving treatment in only one eye, while 41.2% of the patients were receiving treatment in both eyes. The number of previous injections was 12.8 ± 12 (mean ± SD), with a minimum of no previous injections and a maximum of 53. Only 7 patients (6.8%) were naïve. Regarding the type of treatment administered, 52% of patients were being treated with ranibizumab, 27.4% with bevacizumab, and 20.6% with aflibercept.

The mean binocular BCVA at the beginning of treatment, in decimal scale, was 0.53 ± 0.27, and the median level of anxiety measured in mm was 16 (IR: 0–48). Dividing the results into quartiles, 68 patients (66.7%) had a level of anxiety below 25 mm, while 14.7% had levels of anxiety over 50 mm (Table 1).

Furthermore, 27.5% of the patients declared to be not anxious at all (they marked 0 mm in the VASA). In the subgroup of naïve patients, we detected 2 patients (28.6%) with high or very high levels of anxiety (over 50 mm in the VASA). The improvement in binocular BCVA was 0 ± 0.14 (mean ± SD). The real number of injections and the subjective number of injections received were collected, but no significant differences were found between both variables (*p* value = 0.1662).

In univariate models, we found a statistically significant association between anxiety levels and sex. Women were found to have, on average, 10.8 mm more of anxiety levels than men (*p*=0.03), and patients treated with aflibercept had, on average, 14.6 mm less of anxiety levels compared to those treated with other anti-VEGF (*p*=0.046). No statistical significance was found between anxiety levels and the following variables: age, initial binocular BCVA, improvement of binocular BCVA, treatment naïve vs. treatment experienced, real number of injections received, subjective number of injections received (as declared by the patient in the survey), changes in treatment since the beginning of the illness, number of eyes treated, and treated disease.  Table 2 displays the results analyzed.

After including in the multivariate model, all the variables with a value of *p* < 0.15 in the univariate analysis (age, sex, initial binocular BCVA, anti-VEGF, and improvement of binocular BCVA) and eliminating variables by the backward method, the final fitted multivariate model was obtained (Table 3). In this final model, we observed that women presented higher levels of anxiety than men (*p*=0.013), specifically more than 11.87 mm on average. We also found that age was statistically significant (*p*=0.036), and anxiety decreases 4.20 mm on average for every decade. In other words, younger patients had higher levels of anxiety than older ones. However, in this model, anti-VEGF treatment and improvement in binocular BCVA did not show a statistically significant association, but both variables were kept in the model as confounding factors.

## 4. Discussion

Different scales and questionnaires can be used to measure and evaluate anxiety, such as the Hospital Anxiety and Depression Scale, the Depression, Anxiety, and Stress Scale [13], the State-Trait Anxiety Inventory Questionnaire (STAI), or the Amsterdam Preoperative Anxiety and Information Scale (APAIS) [14, 15]. All of them are useful and validated for clinical practice, but they entail sometimes long and harder to perform questionnaires. On the other hand, the VASA, is also a valid scale, is much quicker to perform and presents a good correlation with other validated questionnaires, particularly with the STAI [14–16]. This makes it a good tool for daily clinical practice [17].

The VASA has been used since the 1960s. Initially, it was used to measure levels of pain, but later, its use was extended to include measuring quality of life or levels of anxiety as well as other emotional states [16, 17]. The VASA was first employed in small series of patients that were undergoing dental procedures, and afterwards, it was used to evaluate multiple different procedures and in extensive series of patients, confirming its validity as a tool for evaluating anxiety [18].

Until today, few studies have been made evaluating anxiety and associated factors in patients receiving intravitreal injections [19–23]. Previous studies correlated anxiety levels with demographic variables and factors related to the injection itself, such as the pain perceived. Only Sii et al. [19] tried to relate anxiety levels with changes in BCVA, evaluating only patients diagnosed with AMD. In our study, different macular pathologies were analyzed using a different but validated scale, the VASA. We tried to find relation between anxiety levels and factors related to the diseases and the treatment. We studied variations between the different pathologies, treatment failure and the need to switch to another anti-VEGF, BCVA at the moment of the injection, and changes in BCVA since the beginning of the treatment.

The factors we found to have a significant association with anxiety levels were age and sex. However, we did not find an association with the change in BCVA or with the number of injections received.

The fact that women and younger patients had higher levels of anxiety points to the fact that it is the individual characteristics of each patient that are associated with this emotion, rather than, for instance, the unfavorable course of the disease, or the need for multiple injections. On the other hand, significant differences between anxiety levels and treatment with aflibercept in relation to other anti-VEGF injections were found. The authors think this is a casual finding.

The percentage of subjects with high or very high anxiety levels was 14.7%. We think it could be a good strategy to offer the VASA scale to all patients who are going to receive an intravitreal injection for the first time, paying special attention to women and younger patients. This could allow us to identify those patients with a tendency to higher anxiety levels and thus assure a greater comfort for them in order to improve compliance. Protocols of intravitreal injections administration are usually very standardized. To our knowledge, the procedure of how injections were administered was similar in mentioned studies, so this should not account for the differences observed with other investigations.

Segal et al. [20] analyzed the data of 225 patients who were asked to complete the VASA, measuring their anxiety prior to receiving an intravitreal injection and afterwards measuring their perceived level of pain. In their results, up to 25% of patients had anxiety levels of over 60 mm, while, in our study, the percentage of patients who marked the higher quartile of anxiety was only 4.9%. They found that there was a positive correlation between the level of anxiety measured by the VASA and the level of perceived pain measured with the same scale. They also conclude that, in their sample, women presented higher anxiety levels than men, a finding that coincides with ours.

In 2018, Sii et al. [19] published a study that evaluated anxiety with the VASA in 53 patients receiving intravitreal injections. They also found a positive correlation between the level of pain and anxiety and also concluded that patients had lower anxiety levels when they came accompanied by a family member or friend at the day of the procedure. In their series, like in Segal et al.'s study [20], 75% of patients had anxiety levels below 60 mm. They consider these to be, in general, low levels of anxiety. However, they do not analyze whether anxiety levels vary with the number of injections received. The objective of our study was to identify whether factors related to the disease itself or related to the treatment could somehow influence the level of anxiety, rather than factors related to the particular moment of the injection.

Chaudhary et al., using the STAI, conclude that treatment experienced patients had lower levels of anxiety than those receiving treatment for the first time [21]. In our study, we found no difference between anxiety levels and the number of intravitreal injections received. Kayikcioglu et al. [22], using the STAI scale, and Martel et al. [23], using APAIS scale, did not find differences in anxiety levels in relation to the sex of the patients or to the number of injections received.

Finally, Senra et al. [24], in the qualitative strand of their study, found just over 50% of patients receiving intravitreal injections recognize experiencing some level of anxiety (although they do not quantify it) and do not find a higher percentage of affirmative responses among patients having received three or more intravitreal injections. Results, in general, thus tend to coincide with ours, and we therefore suggest that patients might not get used to the injections and continue to present similar anxiety levels throughout the treatment.

The main limitation of this work is the fact that it is a cross-sectional study; consequently, we cannot draw definitive conclusions about the influence of the evolution of the disease on the anxiety levels. Furthermore, in our sample, only 7 patients were naïve, so we cannot conclude if there is a significant difference between naïve and experienced patients. Future prospective studies should be carried out to corroborate these results.

## 5. Conclusions

The VASA is a valuable tool to measure anxiety in daily clinical practice, due to its accessibility and rapidity. With this scale, we have identified that there is a small percentage of patients with anxiety levels in the highest quartile, and women and younger patients tend to present higher levels of anxiety.

From our point of view, it might be a useful procedure to offer an evaluation of anxiety through the VASA to all patients before they receive their first intravitreal injection, as a screening method to identify the percentage of subjects who suffer a higher level of anxiety. This might lead to further identifying the factors associated with their anxiety and thus offer greater support and assure better treatment compliance.

## Figures and Tables

**Table 1:** Division into four quartiles of the anxiety level measured from 0 to 100 millimeters.

Quartile	Number of subjects	Percentage
0–25	68	66.7
26–50	19	18.6
51–75	10	9.8
76–100	5	4.9

^*∗*^The second row shows the absolute number of subjects that declared that level of anxiety for each quartile. The third row shows the percentage of this number of subjects in relation to the total of subjects (relative frequency).

**Table 2:** Univariate linear regression analysis relating the levels of anxiety with the continuous and categorical variables.

Variable	Coefficient	*p* value
Age (decades)	−3.19	0.116
Sex (female)	+10.79	0.031
Treatment experienced	−13.15	0.152
Number of previous injections	−0.02	0.922
Subjective number of previous injections	−0.09	0.617
Binocular BCVA	+13.96	0.139
Improvement in binocular BCVA	+30.3	0.093
Number of eyes treated (one)	+0.54	0.916
Change in treatment (no)	+4.30	0.392
Anti-VEGF		
Bevacizumab	Reference	
Ranibizumab	−7.11	0.228
Aflibercept	−14.59	0.046
Treated disease		
AMD	Reference	
DME	−0.36	0.947
RVO	−4.51	0.586
Others	+11.83	0.288

BCVA, best-corrected visual acuity; VEGF, vascular endothelial growth factor; AMD, age-related macular degeneration; DME, diabetic macular edema; RVO, retinal vein occlusion.

**Table 3:** Final fitted multivariate regression model.

Variable	Coefficient	*p* value
Age (decades)	−4.20	0.036
Sex (female)	+11.87	0.013
Anti-VEGF		
Bevacizumab	Reference	
Ranibizumab	−9.03	0.136
Aflibercept	−11.88	0.097
Improvement in binocular BCVA	+24.94	0.141

Adjusted *R*^2^ = 0.10. BCVA, best-corrected visual acuity.

## Data Availability

The data used to support the findings of this study are available from the corresponding author upon request
